# Biosimilars: Promises Made. Promises Kept?

**DOI:** 10.1177/12034754211007814

**Published:** 2021-05-26

**Authors:** Sylvia A. Martinez-Cabriales, Rachael Manion, Neil H. Shear

**Affiliations:** 1 12366 Division of Dermatology, Department of Medicine, University of Toronto, Toronto, Ontario, Canada; 2 494622 Department of Dermatology, Sunnybrook Health Sciences Center, Toronto, Ontario, Canada; 3 Canadian Skin Patient Alliance & Canadian Association of Psoriasis Patients, Ottawa, Ontario, Canada

**Keywords:** biosimilars, biologics, therapeutic monoclonal antibodies, drug approval, drug cost, Tumor Necrosis Factor-alpha / antagonists & inhibitors, humira, rituximab

Biologics are a life-changing, high value health proposition for severe dermatologic conditions such as psoriasis, pemphigus, atopic dermatitis, and hidradenitis suppurativa. Biosimilars are facsimiles of biologics promising comparability of therapeutic outcomes while also promising reduced treatment costs to ensure that more patients have access to treatment without impacting the sustainability of the healthcare system. We believe that dermatologists need to actively lobby the government to make sure that these promises are kept.

Biosimilars are considered to have the same efficacy and safety as biologics but are not subjected to rigorous clinical trials. Health Canada evaluates them as new drugs to be certain there are no clinically meaningful differences from the originator biologic. Biosimilar authorization relies on having an appropriately similar therapeutic profile in at least one medical indication to the originator. Additional medical indications are then extrapolated on the basis of the presumption that therapeutic effects will be similar to the originator biologic in all other indications. As of now, no biosimilar is officially deemed interchangeable. Interchangeability, defined as the substitution between equivalent drugs without the prescribing physician’s explicit permission, can be made at the provincial level. Alberta and British Columbia have adopted policies for their publicly funded drug plans to allow for non-medical switching (switching for reasons unrelated to patient health) for certain patients in order to increase biosimilars uptake. Ontario has also announced the forthcoming implementation of this initiative.

Cost savings from the use of biosimilars vary widely and as with generic drugs, the savings might diminish with a decrease in the originator and increase in the similar costs, thus compromising the promised health care systems’ cost savings opportunities and the potential for greater patient access to these therapies. There is no public oversight confirming that cost savings promises are kept and that the actual price of the biosimilars themselves is reduced, rather than savings being achieved by curtailing patient support programs (PSPs). Patient support programs play an important role in patient adherence, which is absolutely necessary for proper care, and are expected from all manufacturers. When a pharmaceutical company provides a biosimilar but fails on the PSP, this is a deterrent to trust. Unprepared PSPs were a significant concern among Canadians switching to biosimilars.

Two significant risks to dermatology patients’ well-being include access to biologics for off-label indications, like pemphigus, and the need for flexible dosing for some medications such as adalimumab for hidradenitis suppurativa. For years, dermatologists have struggled to secure patients’ access to rituximab, which is still off-label, despite being the most effective, cost-effective, and safest treatment for pemphigus. In Ontario, patients have received Rituxan through the provincial Exceptional Access Program, which appears willing to continue to provide Rituxan to patients with an existing Exceptional Access Program approval, and to consider new requests. The comparable biosimilars, Riximyo and Ruxience, have taken over the rituximab market in Ontario, but access to patient support varies dramatically between the two. Requests for approvals and support for patients, among other issues, can delay or deny therapy inappropriately resulting in medical harm (and ironically increased cost). On a national level, the solution for resolving this situation is not clear.

Dermatologists should have a say in biosimilars policies that impact direct patient care. Therefore, on-going attention to outcomes and data collection in the “real world” are needed to better understand biosimilars. In terms of advocacy, the Canadian Association of Psoriasis Patients supports biosimilars in an effort to improve the number of patients with access to therapies for psoriasis and calls on public drug plans to continue to monitor whether patients are faring any worse due to the new biosimilar switching policies. The pan-Canadian biosimilar evaluation framework is one such initiative.

To act in the patient’s best medical interest, dermatologists should collaborate with patient organizations and other stakeholders to ensure patient care is the priority. To facilitate this exchange, we have composed a table of individuals and organizations that should be helpful (Supplemental Table S1). Requesting provincial post-implementation surveillance to assess ongoing responses to biosimilar policies and calling for attention to the need for the off-label use of biosimilars in dermatology practice would help to ensure patients have access to these therapies.

Have promises been met? Time will tell. Vigilance and action are needed. Our patients deserve it.

## Supplemental Material

Table S1 - Supplemental material for Biosimilars: Promises Made. Promises Kept? Click here for additional data file. Supplemental material, Table S1, for Biosimilars: Promises Made. Promises Kept? by Sylvia A. Martinez-Cabriales, Rachael Manion and Neil H. Shear in Journal of Cutaneous Medicine and Surgery

