# Supplementary material for: Biosimilars: Promises Made. Promises Kept?
Source: J Cutan Med Surg. 2021 May 26;25(3):241–2. doi: 10.1177/12034754211007814 (PMC8371028; doi:10.1177/12034754211007814)
Supplement: Table S1 - Supplemental material for Biosimilars: Promises Made. Promises Kept? [file sj-docx-1-cms-10.1177_12034754211007814.docx]

**Supplemental Table**

Biosimilars: Promises made. Promises **kept**?

Martinez-Cabriales SA^1,2^, Manion R^3^, Shear, NH^1,2^

1. University of Toronto, Division of Dermatology, Department of Medicine, Toronto, Canada
2. Department of Dermatology, Sunnybrook Health Sciences Centre, Toronto, Canada
3. Canadian Skin Patient Alliance & Canadian Association of Psoriasis Patients, Ottawa, Canada

| Originator | Biosimilar | Manufacturer/Distributer | Patient Support Programs | Health Canada approval | Medical indications | | | Study population in comparative trials |
| --- | --- | --- | --- | --- | --- | --- | --- | --- |
|  |  |  |  |  | **Moderate to severe Psoriasis** | **PsA** | **Moderate to severe HS** |  |
| Adalimumab  Humira®  AbbVie  AbbVie Care  ‡ AB, MB | Abrilada | Pfizer | X | January 14, 2021 | A PsO | A PsA | A HA  12-17 y (≥ 30 kg) ^ | Phase 1: Healthy M/F  Phase 3: RA despite MTX  therapy |
|  | Amgevita™ (EU)/Amjevita (US)/Solymbic (EU) | Amgen Canada | X | November 4, 2020 | A PsO | A PsA | A HS  12-17 y (≥ 30 kg) ^ | Phase 1: Healthy M/F  Phase 3: RA despite MTX  Therapy  Phase 3: PsO |
|  | Hadlima™/Imraldi | Samsung Bioepis/Merck | X | May 2018 (RA)  March 13, 2019 | A PsO | A PsA | A HS  12-17 y (≥ 30 kg) ^ | Phase 1: Healthy M/F  Phase 3: RA despite MTX  therapy |
|  | Hulio® | BGP Pharma ULC from Mylan S.A.S. | X | November 24, 2020 | A PsO | A PsA | A HS  12-17 y (≥ 30 kg) ^ | Phase 1: Healthy M/F  Phase 3: RA despite MTX  therapy |
|  | Hyrimoz®/Halimatoz/Hefiya | Sandoz | X | November 4, 2020 | A PsO | A PsA | A HS | Phase 1: Healthy M/F  Phase 3: PsO |
|  | Idacio®/Kromeya | Fresenius Kabi | X | October 30, 2020 | A PsO | A PsA | A HS  12-17 y (≥ 30 kg) ^ | Phase 1: Healthy M/F  Phase 3: PsO |
|  | Others biosimilars approved: Cyltezo (Boehringer) FDA approved in PsO and PsA | | | | | | | |
| Humira dosing for PsO: | A: SD: 80 mg sc at wk 0. M: 40 mg qod starting one week after SD. | | | | | | | |
| Humira dosing for HS: | A: SD: 160 mg sc at wk 0, then 80 mg at wk 2. M: 40 mg qw beginning wk 4.  HS & 12-17 y (≥ 30 kg) ^: SD: 80 mg sc at wk 0. M: 40 mg every other wk starting one week after SD, or 40 mg qw depending on patient’s response. | | | | | | | |
| Etanercept  Enbrel®  Immunex Corporation/  Amgen/Pfizer  Enliven®  Ÿ MB | Brenzys®/Benepali (EU)/Eticovo(US) | Merck Canada Inc. | Merck Harmony®  [info@merckharmony.ca](mailto:info@merckharmony.ca) | August 2016 (AS, RA)  August 19, 2020 (Pso, PsA) | A  4-17 y severe PsO* | A PsA | NA | Phase 1: Healthy M only  Phase 3: RA despite MTX  therapy |
|  | Erelzi® | Sandoz | XPOSE®  [erelzi@xposeprogram.ca](mailto:erelzi@xposeprogram.ca) | April 2017 (PJIA, RA)  January 2019 (PsA)  June 2020 (PsO) | A  4-17 y severe PsO*  **‡ AB, MB** | A PsA **‡ AB, BC, MB** | NA | Phase 1: Healthy M only  Phase 3: PsO |
| Etanercept dosing for PsO: | A: SD: 50 mg 2x/wk for 3 months. M: 50 mg qw or 2x/wk  4-17 years with severe PsO*: 0.8 mg/kg per wk (maximum: 50 mg qw) | | | | | | | |
| Infliximab  Remicade®  Janssen's  Janssen BioAdvance®  Ÿ MB | Avsola® | Immunex Corporation/marketed by Amgen | Enliven®  administered/provided by McKesson/Bayshore  [avsola@oneenliven.ca](mailto:avsola@oneenliven.ca) | \| March 12, 2020 \| \| --- \| | A | A PsA | NA | Phase 1: Healthy M/F  Phase 3: RA despite MTX  therapy |
|  | Inflectra®/Remsima SC™ | Celltrion/ Pfizer/ and Celltrion/ Hospira | PfizerFlez™  [www.pfizerflex.ca](http://www.pfizerflex.ca) | January 15, 2014/ January 28, 2021 | A  **‡ AB, BC, MB** | A PsA  **‡ AB, BC, MB** | NA | Phase 1: AS  Phase 3: RA |
|  | Renflexis®(US)/ Flixabi (EU) | Samsung Bioepis/Merck Canada Inc. | Merck Harmony®  [info@merckharmony.ca](mailto:info@merckharmony.ca) | Dec 1^st^, 2017 | A  **‡ AB, BC, MB** | A PsA  **‡ AB, BC** | NA | Phase 1: Healthy M only  Phase 3: RA despite MTX  therapy |
|  | Others biosimilars approved**:** Ixifi (Pfizer) FDA approved /Zessly (Sandoz) EMA approved for Pso + PsA | | | | | | | |
| Infliximab dosing for PsO: | A: SD: 5 mg/kg IV at wk 0, 2, and 6. M: 5 mg/kg q8w | | | | | | | |
| Rituximab  Rituxan®  Roche  Jointeffort® | **Riximyo**™** /Rixathon | Sandoz | XPOSE®  [xpose@sandozprogramsupport.ca](mailto:xpose@sandozprogramsupport.ca) | April 28, 2020 | NA | NA | NA | Phase 1: RA  Phase 3:  Follicular Lymphoma |
|  | Ruxience™ | Pfizer | PfizerFlez™ [ruxience@pfizer.com](mailto:ruxience@pfizer.com) | May 4, 2020 | NA | NA | NA | Phase 1: RA  Phase 3: Low Tumour  Burden Follicular Lymphoma |
|  | Truxima®/Blitzima/Ritemvia/Rituzena | Celltrion Healthcare Co. Ltd./ Teva Canada Limited | Teva Support Solutions® [tss.info@truximacanada.com](mailto:tss.info@truximacanada.com) | April 4, 2019 | NA | NA | NA | Phase 1: Part 1 AFL. Part 2 RA.  Phase 3: RA |
| Biosimilar switching:  ‡ Drug from Tier 1 (AB and MB tiering’s police) or first line therapeutic option by Province unless an exception has been granted. Exception request (medical reason, pregnancy) to prevent switching are being reviewed on a case-by-case basis.  Ÿ Drug from Tier 2  AB: PsO must have failed to 3 drugs (3 different mechanisms of action: Erelzi, Humira, Inflectra, Renflexis; Taltz, Cosentyx; and Skyrizi™) of the Tier 1 before accessing Tier 2 drugs (Stelara). Remicade (since January 15, 2021) and Enbrel (from May 1, 2021) are no longer coverage for dermatology indications^1^.  MB: patients must have failed to 2 drugs from Tier 1 (Erelzi, Humira, Inflectra, Renflexis; Taltz, Cosentyx; Skyrizi™; and Siliq®) before accessing Tier 2 drugs (Enbrel, Remicade, and Stelara)^2^. | | | | | | | | |
| Patient Support Programs: AbbvieCare: Humira®, Skyrizi™ / BioAdvance®: Remicade®, Simponi®, Stelara®, Tremfya® / Enlive: Avsola®, Enbrel®/ Harmony®: Brenzys®, Renflexis®/ LillyPlus™: Taltz® / PfizerFlez™: Inflectra®, Ruxience™ / XPOSE®: Cosentyx®, Erelzi®, Riximyo™. | | | | | | | | |

* Data on safety and efficacy are limited in the age group 4 to 6 years

** Rituximab off-label use in Pemphigus

^ No clinical trials in adolescents. Dosage is based on PK/PD modeling and simulation.

Abbreviations: A: adult, AB: Alberta, Advanced Follicular Lymphoma: AFL, AS: Ankylosing spondylitis, BC: British Columbia, EMA: European Medicines Agency, EU: European Union, F: female, FDA: US Food and Drug Administration, GI: Gastrointestinal, HC: Health Canada, HS: Hidradenitis suppurativa, RA: Rheumatoid arthritis, M: maintenance, Male: male, MB: Manitoba, MPA: Microscopic Polyangiitis, NA: Not applicable, NS: not submitted, PD: Pharmacodynamics, PK: Pharmacokinetic, PJIA: Polyarticular Juvenile idiopathic arthritis, PsA: Psoriatic arthritis, Pso: Psoriasis, SD: Starting dose, US: United States, WG: Wegener’s Granulomatosis, wk: week.

To find more information about each of these medications searching the Health Canada Drug Product Database: <https://health-products.canada.ca/dpd-bdpp/dispatch-repartition.do>

References:

1. Alberta Biosimilar initiative. Biosimilar guide for healthy professionals. Updated December 2020. Page 29. <https://www.ab.bluecross.ca/pdfs/Biosimilar-Health-Professionals-Guide.pdf>.
2. <https://www.gov.mb.ca/health/pharmacare/profdocs/tiered_biologics_specialty.pdf>. Accessed on March 3^rd^, 2021
3. <https://www2.gov.bc.ca/gov/content/health/health-drug-coverage/pharmacare-for-bc-residents/what-we-cover/drug-coverage/biosimilars-initiative-patients> Accessed on March 3rd, 2021
4. <https://health-products.canada.ca/noc-ac/index-eng.jsp>. Accessed on January 23, 2021
5. <https://www.alberta.ca/biosimilar-drugs.aspx>. Accessed on March 3rd, 2021
6. https://health-products.canada.ca/dpd-bdpp/dispatch-repartition.do
